# Entomopathogenic fungi-based mechanisms for improved Fe nutrition in sorghum plants grown on calcareous substrates

**DOI:** 10.1371/journal.pone.0185903

**Published:** 2017-10-05

**Authors:** Silvia Raya-Díaz, Antonio Rafael Sánchez-Rodríguez, José Manuel Segura-Fernández, María del Carmen del Campillo, Enrique Quesada-Moraga

**Affiliations:** 1 Departamento de Ciencias y Recursos Agrícolas y Forestales, Universidad de Córdoba, ETSIAM, Córdoba, Spain; 2 School of Environment, Natural Resources and Geography, Environment Centre Wales, Bangor, United Kingdom; 3 Departamento de Agronomía, Universidad de Córdoba, ETSIAM, Córdoba, Spain; Nederlands Instituut voor Ecologie, NETHERLANDS

## Abstract

Although entomopathogenic fungi (EPF) are best known for their ability to protect crops against insect pests, they may have other beneficial effects on their host plants. These effects, which include promoting plant growth and conferring resistance against abiotic stresses, have been examined in recent years to acquire a better understanding of them. The primary purposes of the present study were (i) to ascertain *in vitro* whether three different strains of EPF (viz., *Metarhizium*, *Beauveria* and *Isaria*) would increase the Fe bioavailability in calcareous or non-calcareous media containing various Fe sources (ferrihydrite, hematite and goethite) and (ii) to assess the influence of the EPF inoculation method (seed dressing, soil treatment or leaf spraying) on the extent of the endophytic colonization of sorghum and the improvement in the Fe nutrition of pot-grown sorghum plants on an artificial calcareous substrate. All the EPFs studied were found to increase the Fe availability during the *in vitro* assay. The most efficient EPF was *M*. *brunneum* EAMa 01/58–Su, which lowered the pH of the calcareous medium, suggesting that it used a different strategy (organic acid release) than the other two fungi that raised the pH of the non-calcareous medium. The three methods used to inoculate sorghum plants with *B*. *bassiana* and *M*. *brunneum* in the pot experiment led to differences in re-isolation from plant tissues and in the plant height. These three inoculation methods increased the leaf chlorophyll content of young leaves when the Fe deficiency symptoms were most apparent in the control plants (without fungal inoculation) as well as the Fe content of the above-ground biomass in the plants at the end of the experiment. The total root lengths and fine roots were also increased in response to fungal applications with the three inoculation methods. However, the soil treatment was the most efficient method; thus, its effect on the leaf chlorophyll content was the most persistent, and the effects on the total root length and fine roots were the most apparent. In conclusion, EPF improved the Fe nutrition of the sorghum plants, but their effects depended on the inoculation method.

## Introduction

Entomopathogenic fungi (EPF) such as *Beauveria*, *Metarhizium* and *Isaria* (Ascomycota: Hypocreales) are commonly found in both agricultural and non-agricultural soils [1]. These fungi are an essential element of most agroecosystems because of their usefulness for agricultural production and pest control in temperate regions [2]. Using EPF for pest control minimizes environmental damage and complies with the stringent laws enacted in recent years (e.g., Regulation (EU) 1107/2009) concerning the placement of plant protection products on the market, in addition, it provides a natural, efficient pest management strategy that is consistent with the concept of sustainable agriculture and the principles of the European Union’s Common Agricultural Policy (CAP).

Entomopathogenic fungi are best known for their microbial control potential; however, they have recently been assigned new roles, especially in relation to their ability to establish themselves as endophytes in the different parts of plants, and to compete with the rest of the rhizosphere microorganisms for root exudates as well as for physical locations near the radicular system [3–5]. In addition, EPFs induce systemic resistance in plants against other biotic stresses such as pathogens and phytoparasitic nematodes [6], promote plant growth [7], increase yields [8], improve plant nutrition [9,10], boost root development [4,11,12] and alleviate abiotic stresses such as salinity [13] or iron (Fe) chlorosis [14,15]. These new ecological functions provide potential additional benefits to plant health while reducing the need for conventional fertilizers, uncovering new horizons [15].

One of these abiotic stresses is Fe chlorosis or Fe deficiency, a nutritional disorder that affects sensitive plants (fruit trees, olive trees, citrus, cereals and berries) grown in calcareous soils [16]. Iron is an essential microelement for plants; thus, it is a component of many enzymatic systems and participates in major processes such as photosynthesis (by catalyzing chlorophyll synthesis) and respiration. The primary symptom of Fe chlorosis is the interveinal yellowing of young leaves due to the inhibition of chlorophyll synthesis and the limited ability by the plants to redistribute Fe in their phloem [17], which may ultimately reduce plant growth and yield [18,19]. Iron is poorly soluble at neutral and alkaline pH values, and it is present as crystalline and amorphous oxides in soil [20]. Crystalline Fe oxides such as goethite and hematite, which prevail in calcareous soils, are less soluble than the more amorphous Fe oxides such as ferrihydrite [21]. As a result, Fe availability in calcareous soils, the pH of which typically ranges from 7.5 to 8.5, is rather low and causes Fe chlorosis in especially sensitive plants [16]. This is a widespread nutritional disorder because calcareous soils account for almost 30% of the world’s arable land area [22,23].

In response to low Fe availability, most fungi develop specific mechanisms to obtain Fe for their own survival [10]. In addition, fungi possess highly efficient Fe acquisition systems such as siderophores. Siderophores are biomolecules with low molecular weights (0.5 to 1.5 kDa) with a high affinity for the Fe^3+^ ion [24–28] that mediates Fe uptake; additionally, they possess Fe reduction mechanisms (ferroxidation and permeation) for the extracellular reduction, and hence the solubilization, of inorganic Fe^3+^ to Fe^2+^ by cell surface metalloreductases [28–31]. Some fungal siderophores act as virulence factors and provide resistance to oxidative stress, thereby facilitating sexual or asexual development, Fe storage and protection from Fe-induced toxicity [28].

Previous studies have revealed that EPF increases the bioavailability of certain nutrients such as Cd, Cu, Pb and Zn [32–34]. Thus, applying *Beauveria bassiana* strain EA04/01-Tip to tomato seeds and wheat grown on artificial calcareous substrates was found to improve Fe nutrition under certain conditions [14], and applying *Metarhizium brunneum* strain EAMa 01/58-Su to the calcareous soils used to pot-grow sorghum, wheat and sunflower improved the Fe bioavailability and/or plant growth [15]. In addition, the inoculation method was found to influence plant growth decisively in bread and durum wheats that were inoculated with *B*. *bassiana* EABb 04/01-Tip when they were grown on a sandy soil [8]. The degree of endophytic establishment and the rhizosphere competence of the EPF have been shown to depend on the particular inoculation method in use (e.g., leaf spraying, seed dressing, and soil treatment) [12, 35–40]; however, there is still a question as to which is the best plant inoculation method for exploiting EPF's potential to increase the Fe bioavailability. The primary purposes of this study were: (1) to ascertain whether applying endophytic strains of *Metarhizium*, *Beauveria* and *Isaria* would modify Fe bioavailability in calcareous and non-calcareous media in an *in vitro* assay with three Fe oxides that differed in their composition, particle size and crystallinity (viz., ferrihydrite, hematite and goethite) and (2) to assess persistence and endophytic colonization by *B*. *bassiana* and *M*. *brunneum*, as previously examined in the *in vitro* assay, and their effects on the growth and Fe nutrition in a sensitive plant such as sorghum grown on an artificial calcareous substrate (a sand mixture) after inoculation with three different methods (seed dressing, soil treatment or leaf spraying).

## Materials and methods

### *In vitro* assay

#### Synthetic iron oxides

Ferrihydrite, hematite and goethite were prepared according to Schwertmann and Cornell [41], Colombo et al. [42] and Torrent et al. [43], respectively, and purified by vigorous stirring, then centrifuged, washed with de-ionized water and dialyzed to a conductivity below 10 μS cm^-1^ to remove the salts. Their specific surface area was determined by using the BET method [44], which is based on N_2_ adsorption measurements and found to be 350 m^2^ g^-1^ for ferrihydrite, 119 m^2^ g^-1^ for hematite and 115 m^2^ g^-1^ for goethite.

**Fig 1:**
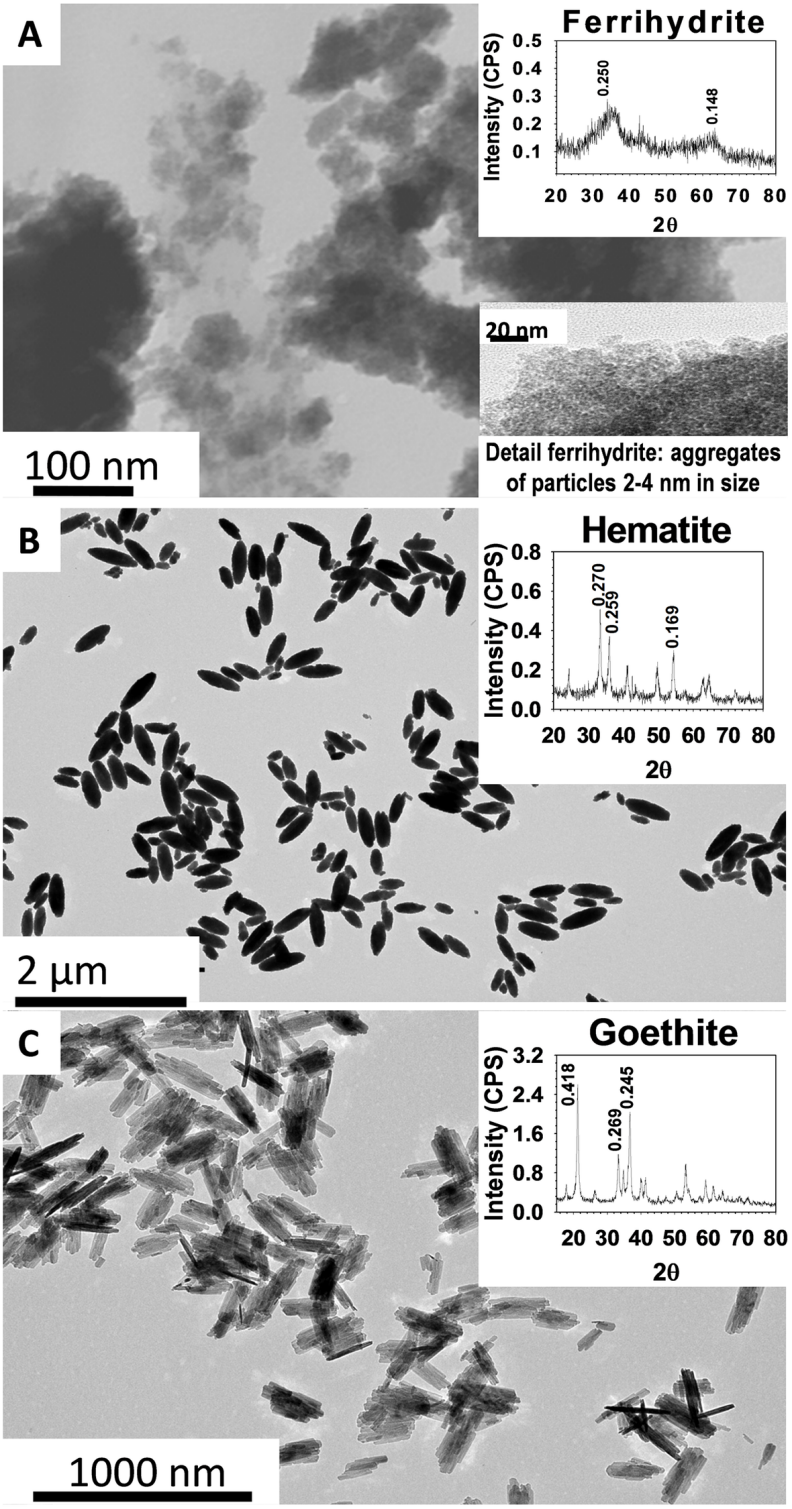
Electron micrographs. Micrographs and characteristic XRD peaks for the Fe oxides. The average particle size of each oxide is shown.

A sample of each Fe oxide was lyophilized and ground in a mortar for analysis on a Siemens D5000 X-ray diffractometer using Co Kα radiation and a JEOL JEM 2010 transmission electron microscope. Fig 1 shows selected electron micrographs of the oxides including their average particle size as well as the characteristic peaks for each oxide in the XRD patterns. The particle size of the oxides was inversely related to their surface area (< 4 nm for ferrihydrite, 100–400 nm for hematite and 100–500 nm for goethite).

#### Fungal strains, experimental design, Fe_DTPA_ and pH analyses

Three different EPF strains that were deposited in the Entomopathogenic Fungi Collection (CRAF) at the University of Córdoba, Spain were used as follows:

1. *Beauveria bassiana* EABb 04/01-Tip isolated from an *Iraella luteipes* larva collected from a field in the town of Carmona (Sevilla, Spain). This strain had previously been found to exhibit endophytic behavior on opium-inoculated plants [45] and it is deposited with accession No. CECT 20744 in the Spanish Collection of Culture Types (CECT) at the University of Valencia (Spain).
2. *Metarhizium brunneum* EAMa 01/58-Su isolated from soil from the town of Hinojosa del Duque (Córdoba, Spain). The specimens were deposited under accession No. CECT 20764 in the Spanish Collection of Culture Types (CECT) at the University of Valencia (Spain).
3. *Isaria farinosa* EAIf 10/01-Msp was isolated from a *Monochamus* (Coleoptera: Cerambycidae) specimen of an unknown species.

The three strains were grown in Petri dishes containing Sabouraud Dextrose Agar (SDA; Oxoid Ltd., Basingstoke, UK) at 25°C in the dark for 15 days, which facilitated optimal growth for fungal sporulation. The mycelia were then carefully removed by scraping the surface of the dishes and placed in sterile beakers containing 50 ml of sterile de-ionized water with Tween 80 (0.1% v/v). The three suspensions (one per fungal strain) were stirred, sonicated for 2 min, filtered to remove the mycelia and adjusted to a concentration of 5 × 10^8^ conidia ml^-1^ by using a hemocytometer (a Malassez chamber). The conidial viability was checked before preparing the suspensions, using germination tests in liquid Czapek-Dox broth containing 1% (w/v) yeast extract. The germination rates exceeded 90% in all the tests.

Then, 0.1 ml aliquots of each fungal suspension were homogeneously distributed over the surface of Petri dishes containing 20 ml of Czapek-Dox solid medium (3 g NaNO_3_, 1 g KH_2_PO_4_, 0.5 g KCl, 0.5 g MgSO_4_ 7 H_2_O, 30 g glucose and 15 g of agar per liter) supplemented with three different Fe oxides (250 mg Fe L^-1^ plus a control containing 0 mg Fe L^-1^) and calcium carbonate (0 or 300 mg CaCO_3_ L^-1^). The control medium was prepared similarly, using 0.1 ml of a fungus-free solution of Tween 80 (0.1% v/v) per Petri dish. A 20 ml volume of Czapek-Dox medium ensured optimal fungal growth and nutrient use during the cultivation period.

After 35 days of cultivation, the fungal mycelium was carefully removed by surface scraping, and fungus-free medium was cut into small pieces (1–2 mm) with sterilized scissors. The pH was measured in a 1 M KCl solution (1:2.5 w/v) and the Fe content was determined on an AAnalyst 200 atomic absorption spectrophotometer from Perkin Elmer (Perkin Elmer AAS) after extraction with 0.005 M diethylenetriaminepentaacetic acid (DTPA, 1:2 w/v), with stirring at 120 rpm for 2 h and centrifugation at 4053 g. The Fe_DTPA_ was used as a measure of the labile Fe [46].

In summary, four fungal treatments (with *M*. *brunneum*, *B*. *bassiana*, *I*. *farinosa* and a control without fungus), three different Fe oxides (250 mg of Fe L^-1^ ferrihydrite, hematite, or goethite, and 0 mg of Fe L^-1^ as a control), and the presence or absence of CaCO_3_ (0 or 300 mg CaCO_3_ L^-1^) were used to develop a completely randomized design with four replicates per combination of the three factors (i.e., 32 treatment combinations, with one Petri dish as the experimental unit and 128 Petri dishes in total).

### Pot experiment

#### Artificial substrate

A mixture containing 71% silica sand as inert medium, 4% silica sand coated with ferrihydrite (Fe oxide-coated sand, FOCS) and 25% calcareous sand to mimic calcareous conditions was used as the substrate. The silica sand was sieved (0.2–0.5 mm), washed several times with Na_2_CO_3_-enriched water (pH 9.5) to disperse clay and impurities, repeatedly washed with de-ionized water and finally dried in an oven with forced aeration at 40°C. The specific surface area of the sand as measured by N_2_ adsorption (BET method) was 0.14 m^2^ g^-1^; its content of citrate/bicarbonate/dithionite-extractable Fe (Fe_d_), which is a measure of total Fe oxides [47], was 40 mg kg^-1^; and its content of available phosphorus as determined by Olsen method [48] was 0.3 mg kg^-1^. Part of the silica sand was coated with ferrihydrite according to Rahmatullah and Torrent [49]. For the FOCS, the Fe_d_ was 380 mg kg^-1^, citrate/ascorbate-extractable Fe (Fe_ca_) [50] at 250 mg kg^-1^ and oxalate-extractable Fe (Fe_ox_) [51] at 210 mg kg^-1^. The Fe_ca_ and Fe_ox_ are acceptably accurate proxies for poorly crystalline Fe oxides, which act as sources of readily available Fe for plants.

The calcareous sand was sieved (0.2–0.5 mm), washed six times with water and once with de-ionized water to remove impurities, and dried at 40°C. It had a surface area of 0.27 m^2^ g^-1^, Fe_d_ = 120 mg kg^-1^, Fe_ca_ = 105 mg kg^-1^ and Olsen P = 1.0 mg kg^-1^.

#### Plants and culture

Cylindrical PVC pots that were 12 cm high and 5 cm in diameter were covered with aluminum foil, and the drainage holes at the bottom were filled with 250 g of the above-described mixture of sands after autoclaving them at 121°C for 20 min twice. Seeds of uniform size from *Sorghum bicolor* L. cv 03CS780/779 were disinfected with 5% sodium hypochlorite for 2 min and then washed several times with sterile de-ionized water. After that, 10 seeds were placed on Petri dishes containing Malt Agar from Biolife Italiana (Milan, Italy) to check the efficacy of the disinfection method. As expected, no fungi or bacteria were re-isolated from the disinfected seeds. Four seeds per pot were then sown before (leaf spraying) or after the fungal applications (seed dressing and soil treatment). All the plants except one in each pot were harvested and removed 20 days after sowing (DAS).

The sorghum crop was held in a growth chamber under photosynthetically active radiation at 350 μmol m^2^ s^-1^, with a photoperiod of 16 h day/8 h night, a day/night temperature of 24/20°C and a relative humidity of 75% for 93 days. The plants were irrigated with 10 ml of modified Hoagland solution per pot on a weekly basis. The solution contained 5 mM Ca(NO_3_)_2_ 4H_2_O, 1 mM KH_2_PO_4_, 5 mM KNO_3_, 2 mM MgSO_4_, 0.05 μM KCl, 25 μM H_3_BO_3_, 2 μM MnSO_4_ H_2_O, 2 μM ZnSO_4_ 7H_2_O, 0.5 μM CuSO_4_·5H_2_O and 3 μM Na_2_MoO_4_·H_2_O. The KH_2_PO_4_ concentration was increased to 10 mM after 2 weeks because some plants exhibited signs of P deficiency, with purple spots on some leaves. The plants were weighed and irrigated to 85% field capacity with de-ionized water on a daily basis, and with nutrient solution once a week.

#### Inoculation methods (treatments) and experimental design

Suspensions containing 5 × 10^8^ conidia ml^-1^ of either *B*. *bassiana* or *M*. *brunneum* were prepared by following the above-described procedure, and they were used separately with the different inoculation methods. These fungal strains were selected in response to the results of the initial assay. The treatments involved applying the fungal solutions in three different ways, and a control treatment without fungus was also used, as follows:

1. *Seed dressing*. This method was used before the seeds were transferred to the pots. A total of 40 seeds were immersed in an aseptic vessel containing 40 ml of fungal suspension, stirred at 120 rpm for 4 h and dried for less than 15 min in a flow chamber before sowing. The seeds used in the other treatments were stirred for 4 h in aseptic vessels containing 40 ml of sterile de-ionized water with Tween 80 (0.1% v/v, no fungus).
2. *Soil treatment*. A 5 ml volume of fungal solution was applied to the top of the artificial substrate in each pot after sowing. All the other pots were supplied with 5 ml of de-ionized water containing Tween 80 (0.1% v/v, no fungus).
3. *Leaf spraying*. This treatment involved spraying 1 ml of fungal suspension onto the first two leaves by using a manual hand sprayer A model 27085 piston compressor from Artesania Latina, (Cantabria, Spain) (23 L min^-1^, 103–345 kPa, 0.3 mm nozzle diameter) was used 26 days after sowing (DAS) and prior to spraying, and the surface of the pots was covered with aluminum foil to prevent the conidia from reaching the substrate. Immediately after the application, the plants were covered with a transparent plastic bag for 24 h to facilitate moisture retention and leaf penetration by the desired fungi. All the other plants were sprayed with 1 ml of de-ionized water containing Tween 80 (0.1% v/v, no fungus) and covered individually with plastic bags for 24 h.
4. *Control method*. In this method, the seeds, substrate and first two leaves were handled similarly to the previous one except that they received a fungus-free solution (Tween 80, 0.1% v/v).

A total of 70 pots were used [(10 pots per combination of inoculation method (soil treatment, seed dressing or leaf spraying) × 2 fungi (*B*. *bassiana* or *M*. *brunneum*), and 10 pots for the control treatment without fungus]. A completely randomized design with four factors (three inoculation methods and a control method) and 10 replicates (pots) per factor was developed for each fungus. The experimental unit was a pot bearing a plant.

#### Substrate analyses: Colony-forming units (CFU) and Fe_DTPA_

A 1 g quantity of substrate was collected from the top 0–2 cm of soil from three randomly selected pots per sampling and inoculation method at 7, 30, 68 and 93 DAS, and also from the control pots. Then, 10 ml of sterile de-ionized water was added to the soil samples and the mixtures were shaken on a Model 3000445 Orbit rotator stirrer from J.P. Selecta (Barcelona, Spain) at 12 rpm for 90 min. Aliquots (0.1 ml) of four different dilutions (1:10, 1:100, 1:1000 and 1:10000) were placed on four Petri dishes containing SDAC (Biolife, Italy). The plates were placed in a culture oven at 25°C for 3–4 days and their numbers of colony-forming units (CFU) were determined by counting.

The fungal growth was visually identified; *B*. *bassiana* colonies exhibited dense white mycelia, whereas *M*. *brunneum* colonies exhibited circular growth, were largely white and contained varying shades of green in the central mycelia [52]. In the event of contamination or potential confusion with other fungal taxa, the mycelia and conidia were removed with a sterile needle and mounted in a drop of fuchsine on a microscope slide. The mounted slide was examined under a Motic BA400 light microscope for typical features of *B*. *bassiana* (viz., globose conidia and zig-zag-shaped conidiophores) and *M*. *brunneum* (conidiophores heavily branched in a candelabrum-like manner but very densely intertwined and forming nearly wax-like fertile areas; short, blocky conidiogenous cells lacking apical necks; and long conidial chains usually laterally adherent in prismatic columns or continuous plates) [52].

Finally, substrate from four pots per inoculation method and fungus was used to determine the Fe_DTPA_ at the end of the experiment. For this purpose, 10 g of soil per pot was shaken with 20 ml of 0.005 M diethylenetriaminepentaacetic acid (DTPA, 1:2 w/v) at 120 rpm for 2 h before centrifugation at 4053 g. The Fe_DTPA_ was determined on the Perkin Elmer AAS as described in the *in vitro* assay section.

#### Fungal re-isolation from plant tissues

The endophytic colonization by *B*. *bassiana* and *M*. *brunneum* was assessed in four determinations at 20, 28, 70, and 93 DAS. Three plants per inoculation method (plus one from the control method) and sampling time were randomly selected and harvested. During the first sampling (20 DAS), the plants were randomly selected from those that were sown at the beginning of the experiment and harvested to leave a single plant per pot (experimental unit). For the second and third samplings (at 28 and 70 DAS, respectively), the total number of pots per treatment and fungal strain was reduced to 7 and 4, respectively. In the last sampling (93 DAS), three of the last four plants per treatment and fungal strain were used to re-isolate the endophytes and all four were used for additional analyses (see next section).

For each sampling, the plants were harvested, and their leaves, stems and roots were separated. A sample of the different plant parts was disinfected in 2% NaClO (5% for roots) for 2 min before washing with sterile de-ionized water twice for 2 min. The effectiveness of the surface sterilization method was tested by plating 100 μL aliquots of 10^−1^ to 10^−3^ dilutions from the water used to wash the plant material in Malt Agar from Biolife Italiana (Milan, Italy) and incubated at 25°C for 2 weeks to determine the CFU. As expected, no fungi or bacteria were re-isolated from the water that was used to wash the plant material. The surface of the plant material was properly sterilized since no fungi or bacteria were re-isolated from the water that was used to wash the plant material.

The two youngest leaves in each sampling were split into 6 fragments of 1 cm^2^ each, and the stems and roots were split into 6 fragments that were 0.5 cm long. The resulting fragments were separately placed on Petri dishes (one dish per plant for leaves, another for stems and a third for roots) containing selective medium for *B*. *bassiana* and *M*. *brunneum*. The *B*. *bassiana* selective medium consisted of 500 ml of de-ionized water containing 10 g of oatmeal infusion, 10 g of agar, 225 mg of dodine (*N*-dodecylguanidine monoacetate), 2.5 mg of chlortetracycline as an antibacterial and 5 ml of a 10 mg L^-1^ solution of Crystal Violet. The *M*. *brunneum* selective medium contained the following components, which were suspended in 500 ml of de-ionized water: 5 g of glucose, 5 g of bacteriological peptone, 7.5 g of ox gall, 17.5 g of agar, 5 mg of dodine (*N*-dodecylguanidine monoacetate), 125 mg of cycloheximide and 250 mg of chloramphenicol as an antibacterial. The Petri dishes containing the different plant tissues were placed in a culture oven at 25°C for approximately 15–20 days, during which the proportion of fragments exhibiting fungal outgrowth was recorded.

#### Plant and root parameters

The plant height, number of leaves and leaf chlorophyll concentration (LCC) in the two youngest leaves from each pot were determined at 20, 29, 34, 42, 49, 56, 72, 79, 87 and 93 DAS. These variables were measured in 10 plants at the beginning of the experiment, but the number was reduced to 4 when they had to be harvested to assess the endophytic colonization in the different samplings (destructive samplings). The LCC was determined with an SPAD 502 portable chlorophyll meter from Minolta Camera Co. (Osaka, Japan). The measurements were validated at the end of the experiment against the chlorophyll total concentration (CTC) extracted from the two youngest leaves with a 99.5 wt% solution of methanol (*r* = 0.75, *p* < 0.001) according to [53]. The SPAD proved a reliable proxy for estimating the CTC in the pot experiment.

The last four sorghum plants for each inoculation method, control method included, and the fungal strain remaining at the end of the experiment (93 DAS) were harvested and split according to the plant tissue (roots, stems and leaves). A portion of each type of tissue was used to assess the fungal re-isolation as described in the previous section. The remaining roots were washed with de-ionized water for scanning on an Epson Perfection V700 scanner, and the captured images were analyzed with WinRhizo^®^ software (Régent Instrument, Inc., Québec, Canada, regent.qc.ca). As recommended by [54], we used the automatic thresholding in the imaging software to optimize the threshold that divided the gray levels into two distinct groups (roots and background) and to determine the root length, specific root length (SRL, Eq 1) [55,56], specific root area (SRA, Eq 2) [57,58] and root length as a function of the root diameter (*L* ≤ 0.2, 0.2 < *L* ≤ 1.0, *L* > 1.0 mm).

$$SRL\left(m/g\right)=\frac{Root\;length}{Dry\;weight}$$ (1)

$$SRA(m^{2}/kg)=\frac{Root\;area}{Dry\;weight}$$ (2)

The leaves and stems (in combination) and roots (after scanning) were dried in an oven at 70°C for at least 73 h to determine the dry weight per plant. The dried leaves were ground and digested with a mixture of nitric and perchloric acids [59]. The Ca, Mg, Fe, Mn, Zn and Cu were determined on the Perkin Elmer AAS, K on a Jenway PFP7 flame emission spectrometer and P by Molybdenum Blue method [60] on a Perkin Elmer Lambda 35 UV/VIS spectrophotometer. The C and N were quantified by direct combustion on an EA3000 Analyzer from Eurovector SpA (Milan, Italy).

#### Statistical analysis

Statistical analyses were performed with STATISTIX 10 software from Analytical Software (Tallahassee, FL, USA). Previously, the data were checked for normal distribution and homoscedasticity by using the Kolmogorov-Smirnov test and Levene’s test, respectively. An analysis of variance (ANOVA) was performed to identify the effects of the studied factors (fungal strain, Fe oxide and CaCO_3_) on the pH and Fe_DTPA_ in the *in vitro* assay and on the dry weight of the above-ground biomass and roots; nutrient contents of above-ground biomass; CTC; root length; SRL, SRA and root length as a function of root diameter in the sorghum plants; CFU in soil and re-isolation of each fungal strain from plant tissues in the pot experiment. An additional factorial ANOVA for the Fe_DTPA_ and pH was performed for each combination of fungal strain, Fe oxide and the absence or presence of CaCO_3_ in the *in vitro* assay. Logarithmic transformations were applied whenever the requirements for parametric analyses were not met. Additional correlation analyses between the pH and Fe_DTPA_ at the end of the experiment (35 days) were performed. In addition, a split-plot ANOVA was performed on each fungal strain with a provision for the factor time and the interaction time × inoculation method for the plant height, number of leaves, LCC and LCC × plant height for each fungal strain. When the differences were significant (*p* < 0.05), a *post hoc* LSD test was used to separate the means. The control group was excluded from the statistical analyses for CFU in soil and the colonization of plant tissues in the pot experiment because it almost invariably had zero values.

## Results

### *In vitro* experiment

Table 1 shows the results of the factorial analysis for the Fe_DTPA_ and pH in the Czapek-Dox medium at the end of the *in vitro* assay (after 35 days). Regarding Table 1, *M*. *brunneum* could be said to produce the highest Fe_DTPA_ concentrations (in mg Fe L^-1^), at 27.2 ± 5.6 and was followed in this respect by *I*. *farinosa* (12.3 ± 1.8), *B*. *bassiana* (9.7 ± 1.9) and the control without fungus (1.2 ± 0.3). Table 1 indicates that ferrihydrite was the individual Fe oxide leading to the highest Fe_DTPA_ contents in the medium at 30.1 ± 5.2, followed by hematite (10.8 ± 1.9) and goethite (9.2 ± 1.9). However, the addition of CaCO_3_ decreased the Fe_DTPA_. *Beauveria bassiana* and *I*. *farinosa* raised the pH of the medium (to 8.1 ± 0.1 and 8.2 ± 0.1, respectively); by contrast, *M*. *brunneum* did not alter the pH relative to the control without fungus (6.5 ± 0.1 vs 6.7 ± 0.3). Finally, all three Fe oxides increased the pH in relation to the control without Fe (7.4–7.5 vs 7.0 ± 0.3), and so did the presence of CaCO_3_ (7.9 ± 0.1–6.8 ± 0.2).

**Table 1:** Factorial ANOVA for the Fe_DTPA_ and pH (mean and standard error, *n* = 4) of the Czapek-Dox medium after culturing the three strains at 25°C for 35 days with each combination of fungus, Fe oxide (0 or 250 mg L^-1^) and the presence or absence of CaCO_3_.

	Fe_DTPA_ (mg L^-1^)	pH_KCl_
**Fungus**		
No fungus	1.2 ± 0.3	6.7 ± 0.3
*B*. *bassiana*	9.7 ± 1.9	8.1 ± 0.1
*I*. *farinosa*	12.3 ± 1.8	8.2 ± 0.1
*M*. *brunneum*	27.2 ± 5.6	6.5 ± 0.1
*p value*	< 0.001	< 0.001
**Fe oxide**		
Control^(a)^	0.0 ± 0.0	7.0 ± 0.3
Ferrihydrite	30.1 ± 5.2	7.5 ± 0.2
Hematite	10.8 ± 1.9	7.5 ± 0.2
Goethite	9.2 ± 1.9	7.4 ± 0.2
*p value*	< 0.001	< 0.001
**CaCO**_**3**_		
0 mg L^-1^	17.0 ± 3.1	6.8 ± 0.2
300 mg L^-1^	8.1 ± 1.3	7.9 ± 0.1
*p value*	< 0.001	< 0.001
**Interactions**		
Fungus × Fe oxide	<0.001	<0.001
Fungus × CaCO_3_	0.045	<0.001
Fe oxide × CaCO_3_	0.096	<0.001
Fungus × Fe oxide × CaCO_3_	<0.001	0.449

^(a)^ Control (0 mg Fe L^-1^)

**Fig 2:**
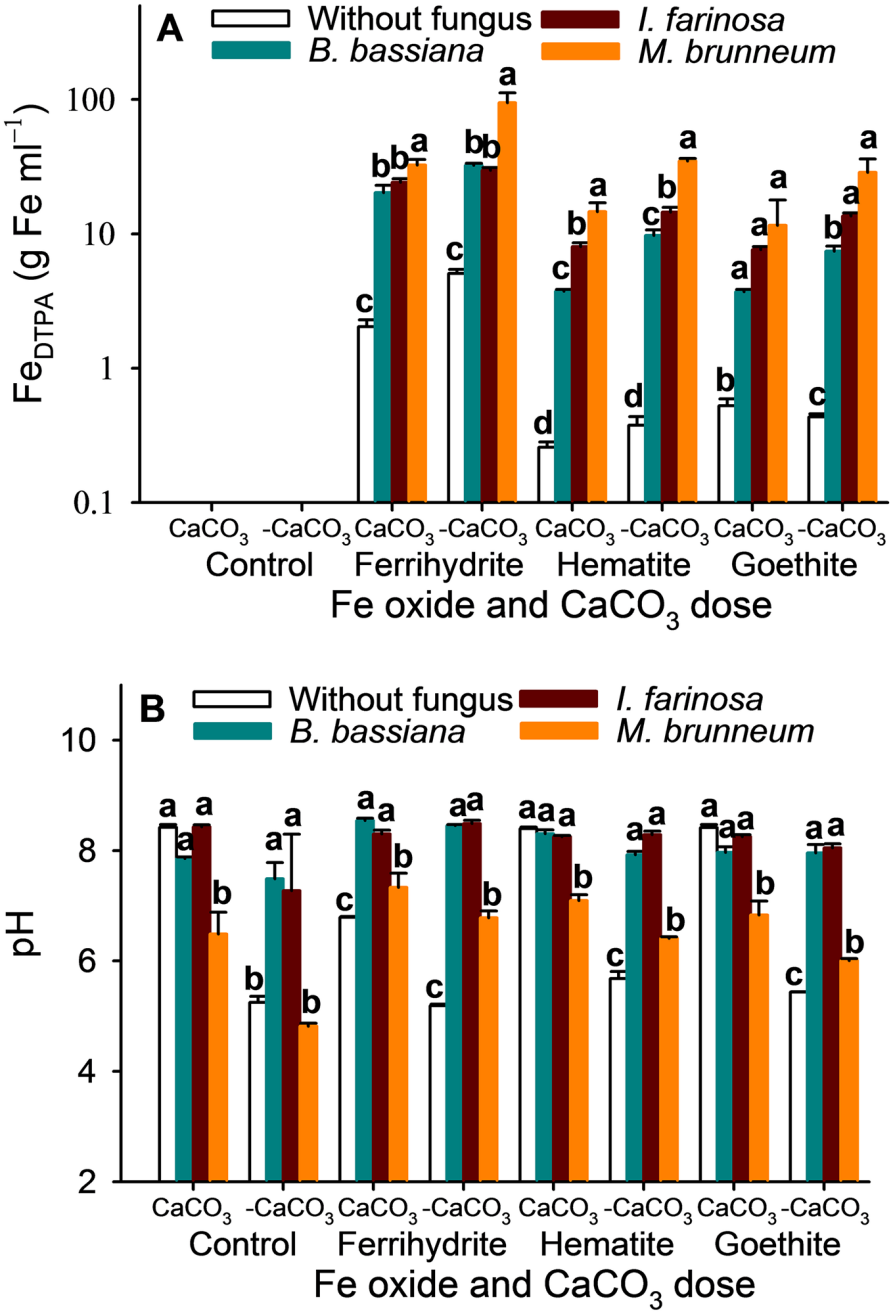
Mean Fe_DTPA_ and pH. The Fe_DTPA_ (A) and pH (B) in the Czapek-Dox medium (mean ± standard error, *n* = 4) at the end of the *in vitro* assay. Different letters indicate significant differences between the different levels of each factor according to an LSD *post hoc* test at *p* < 0.05.

In any case, there were multiple interactions between the different factors for the Fe_DTPA_ and pH except for Fe oxide × CaCO_3_ in Fe_DTPA_ (*p* = 0.096) and Fungus × Fe oxide × CaCO_3_ and pH (*p* = 0.449) (see Table 1). These interactions are illustrated in Fig 2 and the S1 Table. The S1 Table indicates that the presence of CaCO_3_ decreased the Fe_DTPA_ and increased the pH in most determinations. In all the factor combinations, the EPF activity led to increased Fe_DTPA_ values in relation to the control without fungus. Although *M*. *brunneum* invariably produced the highest Fe_DTPA_ concentrations, the differences from the other two fungi with goethite as the Fe source in the presence of CaCO_3_ were not significant, nor were those between *M*. *brunneum* and *I*. *farinosa* with the same Fe source in the absence of CaCO_3_ (Fig 2A). In addition, *I*. *farinosa* significantly increased the Fe_DTPA_ in relation to *B*. *bassiana* with hematite as the Fe source, whether or not CaCO_3_ was present, and with goethite in the absence of CaCO_3_. However, this trend was not found with the other combinations of factors (Fig 2A). The fungus-free treatment led to the lowest pH values in the absence of CaCO_3_ except when no Fe source was used; however, it exhibited the opposite trend with ferrihydrite as the Fe source in the presence of CaCO_3_ (Fig 2B). Overall, *I*. *farinosa* and *B*. *bassiana* increased the pH compared to *M*. *brunneum* in all the combinations of factors. In addition, *M*. *brunneum* resulted in the lowest pH in the presence of CaCO_3_, except with ferrihydrite, as shown in S1 Table and Fig 2B.

The Fe_DTPA_ and pH were negatively correlated in the control without Fe in the absence of CaCO_3_ (*r* = –0.47, *p* < 0.001). Conversely, these two variables were positively correlated for *B*. *bassiana*, *I*. *farinosa* and *M*. *brunneum* (*r* = 0.72, *r* = 0.36, and *r* = 0.80, respectively; *p* < 0.001). In the presence of CaCO_3_, the pH and Fe_DTPA_ were negatively correlated in the dishes without fungus (*r* = –0.94, *p* < 0.001), but positively correlated in those containing *B*. *bassiana*, *I*. *farinosa* or *M*. *brunneum* (*r* = 0.74, *r* = 0.35, *r* = 0.63, respectively; *p* < 0.001).

### Pot experiment

#### Colony-forming units (CFU)

**Fig 3:**
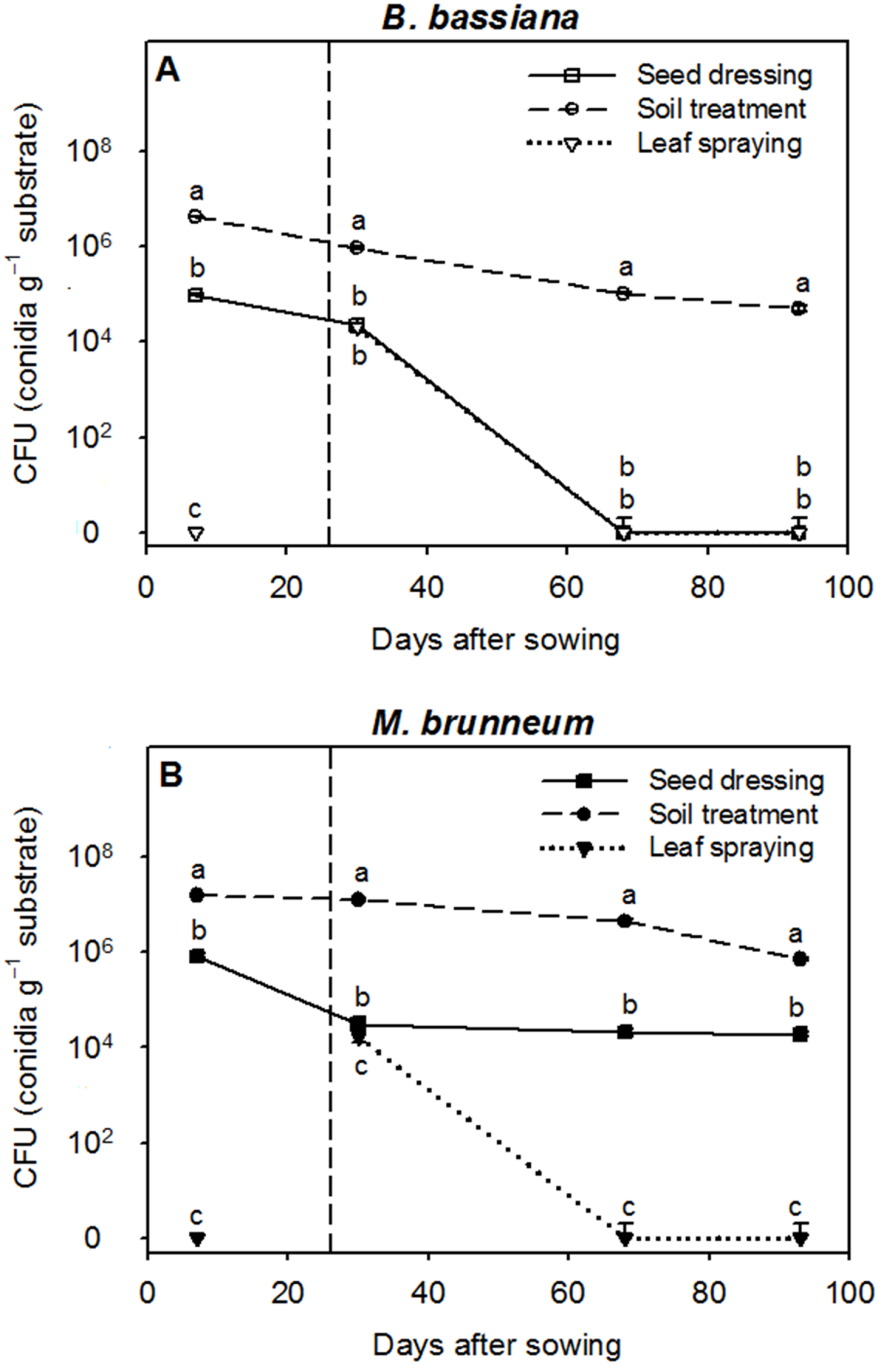
Colony-forming units (CFU). The number of conidia g^-1^ in the calcareous substrate (mean ± standard error, *n* = 4) for each fungal strain as a function of the inoculation method (seed dressing, soil treatment and leaf spraying) at 7, 30, 68 and 93 days after sowing (DAS). The dashed line indicates the time of application of the leaf treatment. No *B*. *bassiana* or *M*. *brunneum* CFUs were detected in the substrate samples from the control pots. Different letters indicate significant differences between inoculation methods in each sampling according to the LSD *post hoc* test at *p* < 0.05.

Fig 3 shows the time course of CFU in the substrate. As expected, no *B*. *bassiana* or *M*. *brunneum* CFU were detected in the substrate samples from the control pots. The highest CFU concentrations in the first sampling (7 DAS) were those in the substrates from the soil treatment (4.3 × 10^6^ ± 3.3 × 10^4^ conidia g^-1^ for *B*. *bassiana* and 1.6 × 10^7^ ± 3.4 × 10^5^ conidia g^-1^ for *M*. *brunneum*), followed by seed dressing (9.7 × 10^4^ ± 3.3 × 10^3^ conidia g^-1^ for *B*. *bassiana* and 8.3 × 10^5^ ± 8.8 × 10^4^ conidia g^-1^ for *M*. *brunneum*) and leaf spraying (no CFU detected, *p* < 0.001). In the second sampling, which was performed four days after leaf spraying (i.e., 30 DAS), the soil treatment led to significantly (*p* < 0.001) higher CFU concentrations (9.3 × 10^5^ ± 6.7 × 10^4^ conidia g^-1^ for *B*. *bassiana* and 1.3 × 10^7^ ± 1.1 × 10^6^ conidia g^-1^ for *M*. *brunneum*) than the seed dressing and leaf spraying (*ca*. 2 × 10^4^ conidia g^-1^ for both treatments and fungi). A decrease in the CFU was observed with both fungi that was especially sharp with seed dressing and leaf spraying in *B*. *bassiana* (Fig 3A), and with leaf spraying in *M*. *brunneum* (Fig 3B), during the third sampling (68 DAS). Note that seed dressing resulted in a constant CFU concentration with *M*. *brunneum* throughout the experiment and that leaf spraying only resulted in CFU production at 30 DAS with both fungal strains.

#### Fungal re-isolation

**Fig 4:**
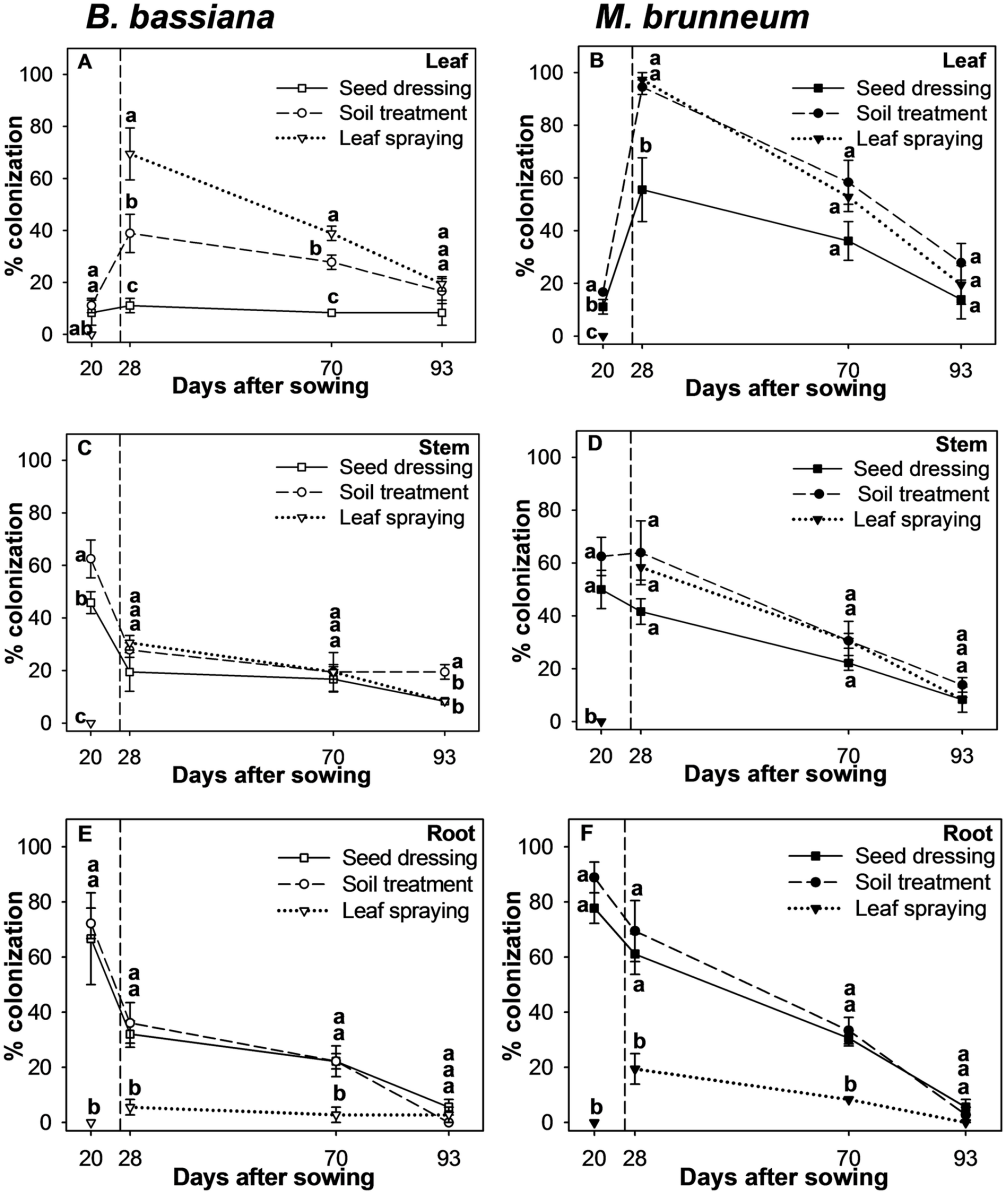
Re-isolation. The time course for the colonization (mean ± standard error, *n* = 4) of the leaves, stems and roots by *B*. *bassiana* (A, C, E) and *M*. *brunneum* (B, D, F) as a function of the inoculation method. The dashed line indicates the time of the leaf spraying (26 DAS) application. Different letters indicate significant differences between inoculation methods according to the LSD *post hoc* test at *p* < 0.05.

Neither *B*. *bassiana* nor *M*. *brunneum* were detected in any plant tissue from the control plants. In addition, neither fungus was re-isolated from the leaves, stems or roots of the leaf-sprayed plants in the first sampling (20 DAS) because this treatment was applied 6 days after sampling (26 DAS). The fungal re-isolation from the different plant tissues progressively decreased after the second sampling until the end of the culture (93 DAS), independent of the inoculation method (Fig 4).

The re-isolation of both fungi from leaves peaked in the second sampling (28 DAS) and amounted to 69 ± 10% with leaf spraying, 39 ± 7% with soil treatment and 11 ± 3% with seed dressing in *B*. *bassiana* (Fig 4A), 90% with leaf spraying and soil treatment with no difference between the two and 56 ± 12% with seed dressing in *M*. *brunneum* (Fig 4B). A decrease in re-isolation was observed in the third and fourth samplings (70 and 93 DAS, respectively) with leaf spraying (39 ± 3% and 19 ± 3%) and soil treatment (28 ± 3% and 17 ± 5%). However, the re-isolation of *B*. *bassiana* as applied by seed dressing remained constant at 8 ± 5% (Fig 4A). A decrease in the *M*. *brunneum* re-isolation was also observed in the third and fourth samplings with the leaf spraying (53 ± 6% and 19 ± 7%), soil treatment (58 ± 8% and 28 ± 7%) and seed dressing (36 ± 7% and 14 ± 7%) (Fig 4B). However, the differences among treatments at the end of the experiment were not significant (Fig 4A and 4B).

During the first sampling (20 DAS), soil treatment resulted in the highest re-isolation (63 ± 7%), followed by seed dressing (46 ± 4%) in *B*. *bassiana* (Fig 4C). During the second and third samplings (28 and 70 DAS, respectively), a decrease in re-isolation was observed with leaf spraying (31 ± 3% and 19 ± 7%) and soil treatment (28 ± 3% and 19 ± 3%); this was not the case with seed dressing (19 ± 7% and 17 ± 5%), which resulted in insubstantial differences among the treatments (Fig 4C). The previous values are lower than those obtained with seed dressing and soil treatment in the first sampling. After the fourth sampling (93 DAS), the soil treatment resulted in greater re-isolation (19 ± 3%) than leaf spraying and seed dressing (*ca*. 8 with both) (Fig 4C). The re-isolation of *M*. *brunneum* at 20 DAS (Fig 4D) exhibited 63 ± 7% with soil treatment and 50 ± 7% with seed dressing, with no significant differences between the two. In the second and subsequent samplings, the differences among treatments were not significant. The re-isolation decreased in the following sequence from 28 to 93 DAS: soil treatment (64 ± 12% to 14 ± 3%) > leaf spraying (58 ± 5% to 8 ± 5%) > seed dressing (42 ± 5% to 8 ± 0%) (see Fig 4D).

Significant differences in root colonization among treatments were observed in the first, second and third samplings (Fig 4E and 4F). The seed dressing and soil treatment showed no significant differences between the two and resulted in the greater re-isolation of both fungi from 20 to 70 DAS than did leaf spraying. The re-isolation declined gradually in the following sequence with *B*. *bassiana*: soil treatment (72 ± 6% to 22 ± 6%) > seed dressing (67 ± 17% to 22 ± 3%), with no significant differences between the two (Fig 4E). The situation was similar for *M*. *brunneum*, with no significant differences between the soil treatment (a decrease from 89 ± 6% to 33 ± 5%) and seed dressing (78 ± 6% to 31 ± 3%) (Fig 4F). The re-isolation with leaf spraying never exceeded 6 ± 3% for *B*. *bassiana* or 19 ± 6% for *M*. *brunneum* in any sampling.

#### Plant growth and LCC

**Fig 5:**
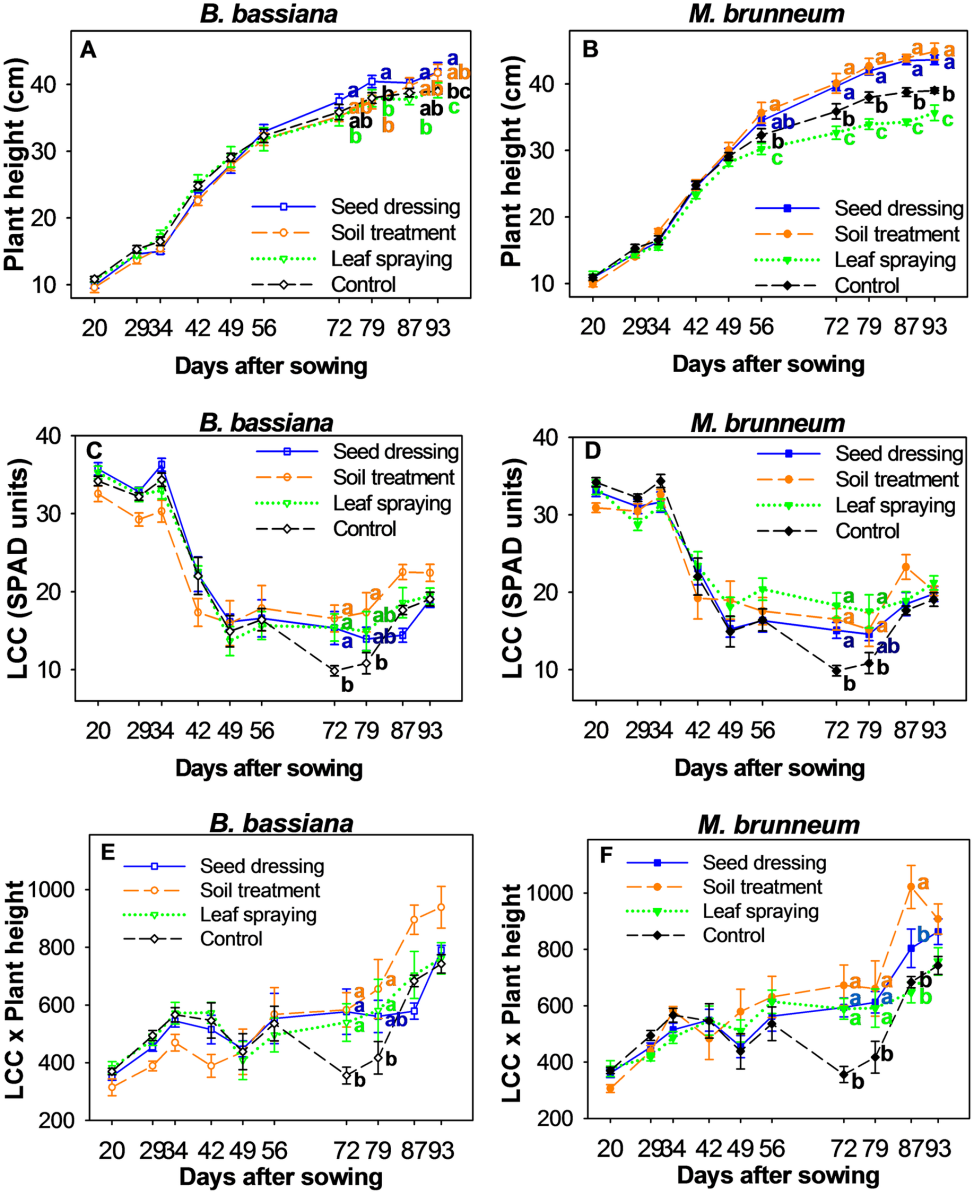
Plant height, LCC and LCC × Plant height in sorghum. A time course of the sorghum plant height, LCC (leaf chlorophyll concentration) and LCC × plant height (mean ± standard error, *n* = 4) for *B*. *bassiana* (A, C, E) and *M*. *brunneum* (B, D, F) as a function of the inoculation method. Different letters indicate significant differences between inoculation methods according to the LSD *post hoc* test at *p* < 0.05.

As observed in Fig 5A and 5B, the fungal inoculation method used here influenced the sorghum plant height. Thus, from 72 DAS to 93 DAS, the *B*. *bassiana* plants inoculated by seed dressing were significantly taller than the ones that were inoculated by leaf spraying (Fig 5A). The seed-dressed plants were also significantly higher than those in the soil treatment and control groups at 79 DAS, and higher than the control plants at 93 DAS. In most cases, the leaf-sprayed plants were the shortest, but the differences from the control plants were never significant. The tallest *M*. *brunneum* plants from 72 DAS to the end of the experiment were in the soil treatment and seed dressing groups (*p* < 0.001 for each determination; Fig 5B). Leaf spraying produced the shortest plants in relation to the other treatments (control plants included). Although a similar effect was observed at 56 DAS, no significant differences in this respect were found between the seed dressing and control plants (Fig 5B).

Fig 5C and 5D show the time course of the LCC. As observed, there were three distinct stages. The LCC peaked in the first stage (20 to 34 DAS), at 33.6 ± 0.6 in the control plants, 33.1 ± 0.9 in *B*. *bassiana*-inoculated plants and 31.5 ± 0.8 in *M*. *brunneum*-inoculated plants, with no significant differences between the inoculation methods and the control treatment in either fungus. During the second stage (42 to 79 DAS), the LCC dropped and the Fe chlorosis symptoms (viz., interveinal yellowing) became apparent. There were significant differences among the treatments in inoculated plants at the end of this stage. Thus, the inoculated *B*. *bassiana* plants exhibited significantly higher LCC values than their control counterparts at 72 DAS (*p* = 0.021 for the three inoculation methods) and 79 DAS (*p* = 0.048, but they were significant only in the soil treatment group) (Fig 5C). Inoculated *M*. *brunneum* plants also differed significantly from the control plants at 72 DAS (*p* < 0.001 for the three inoculation methods) and 79 DAS (*p* = 0.013, but significant only with leaf spraying and soil treatment) (Fig 5D). In the last two measurements, the LCC increased slightly and became more similar among treatments, with no significant differences through the end of the experiment (third stage, 87 to 93 DAS).

Fig 5E and 5F show the LCC × Plant height interaction as a measure of the overall plant status regarding Fe chlorosis, interveinal yellowing and growth. All the fungal treatments led to higher values in *B*. *bassiana* than did the control treatment at 72 DAS (*p* = 0.007) and 79 DAS (*p* = 0.013); however, on the latter date, the differences from the control group were only significant with soil treatment and leaf spraying (Fig 5E). In *M*. *brunneum* (Fig 5F), the three fungal treatments led to significantly higher LCC × Plant height values than the control treatment at 72 DAS (*p* < 0.001) and 79 DAS (*p* = 0.005). Subsequently, the only significant differences were those resulting from soil treatment, which led to the highest values, at 87 DAS (*p* = 0.055 for *B*. *bassiana* and *p* < 0.001 for *M*. *brunneum*).

There were no significant differences in the number of leaves between the plants inoculated with *B*. *bassiana* and those in the control group (S1A Fig). By contrast, the plants inoculated with *M*. *brunneum* (S1B Fig) differed significantly among the groups (*p* < 0.001) in all the samplings except at 49 DAS). The smallest numbers of leaves were those in the soil treatment plants at 20, 29, 34, 42 and 72 DAS and those in the plants of the soil treatment, seed dressing and control groups at 56, 79, 87 and 93 DAS. By contrast, leaf spraying produced the largest number of leaves at the end of the cropping period (56 to 93 DAS, S1B Fig).

The CTC values (in μg cm^-2^) of plants inoculated with *B*. *bassiana* at the end of the experiment (93 DAS) decreased in the following sequence (S2 Table: soil treatment > seed dressing > control treatment > leaf spraying (*p* = 0.029). The sequence for plants inoculated with *M*. *brunneum* was as follows: leaf spraying > soil treatment > seed dressing > control treatment (*p* = 0.011).

#### Root parameters

The root dry weights (S3 Table) were unaffected by the fungal strain and inoculation method. However, the total root length (m) for both fungal strains was significantly influenced by the inoculation method. Thus, the total root length in the plants inoculated with *B*. *bassiana* was 21.2 ± 2.4 with seed dressing, 21.6 ± 1.4 with soil treatment and 22.5 ± 2.8 with leaf spraying, with all three values being significantly greater (*p* = 0.031) than that for the control plants (12.2 ± 1.3) but not significantly different from one another. With *M*. *brunneum* (*p* < 0.004), the total root length was greatest with soil treatment (26.5 ± 0.8), followed by leaf spraying (21.3 ± 2.3), seed dressing (18.2 ± 2.7) and the control treatment (12.2 ± 1.3) (S2 Fig).

**Fig 6:**
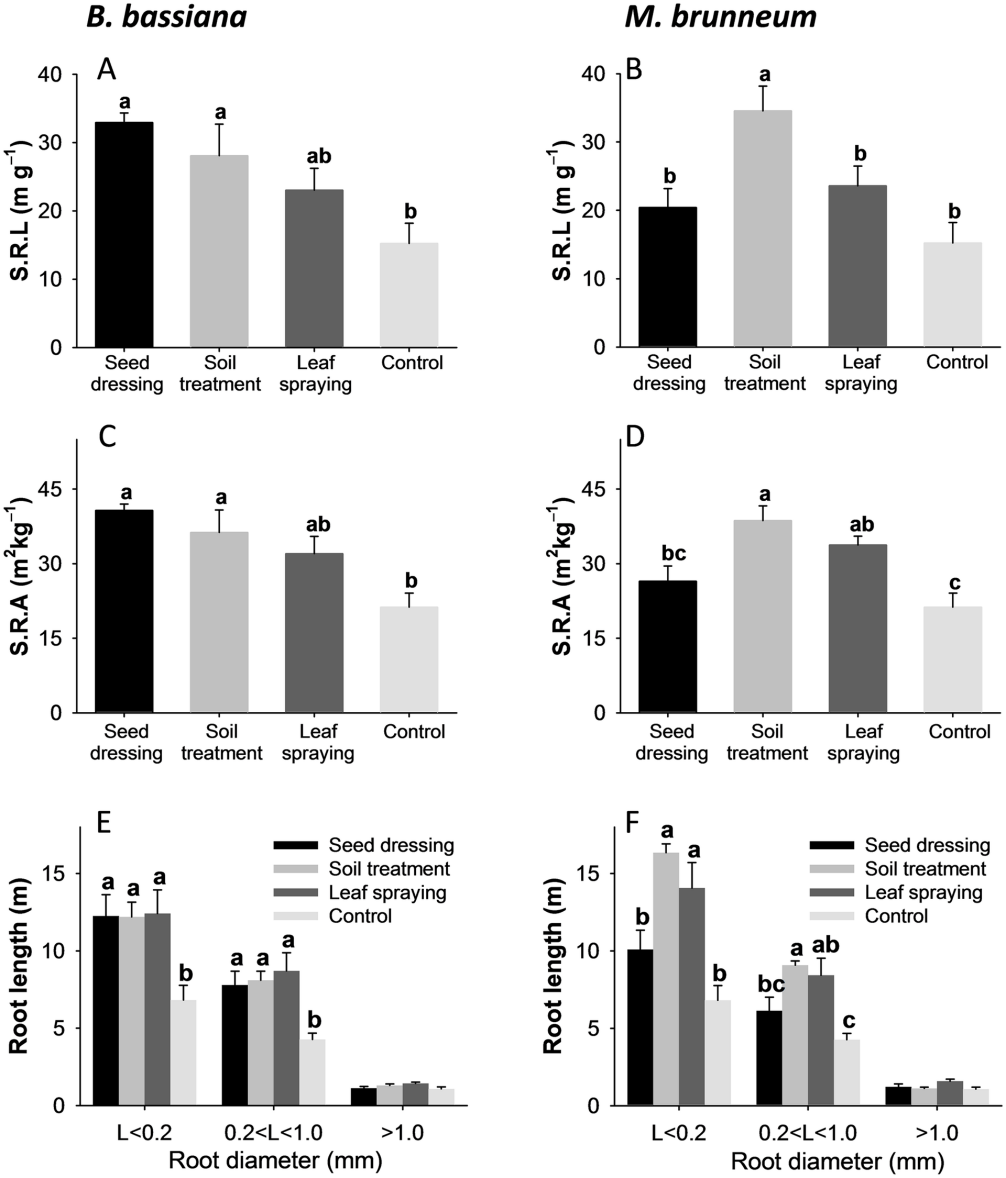
Root parameters. Specific root length (SRL), specific root area (SRA) and root length according to the root diameter (mean ± standard error, *n* = 4) for sorghum plants inoculated with *B*. *bassiana* (A, C, E) or *M*. *brunneum* (B, D, F) by using different inoculation methods. Different letters indicate significant differences between inoculation methods according to an LSD *post hoc* test at *p* < 0.05.

The specific root length (SRL) in the plants inoculated with *B*. *bassiana* (Fig 6A) was greater with seed dressing and soil treatment than with the control treatment (*p* = 0.024); however, there were no significant differences with leaf spraying. A similar trend was observed with *M*. *brunneum* (Fig 6B), but the differences were only significant with the soil treatment (*p* = 0.008). The specific root area (SRA) in the plants inoculated with *B*. *bassiana* exceeded that in the control plants; the differences were significant for all treatments except leaf spraying (*p* = 0.014, Fig 6C). The plants inoculated with *M*. *brunneum* behaved similarly with regards to the SRA, with the differences being significant for all treatments except seed dressing (*p* = 0.005, Fig 6D).

The fine roots (less than 0.2 mm in diameter) were less abundant in the control plants than in those inoculated with *B*. *bassiana* (*p* = 0.039, Fig 6E) or *M*. *brunneum* (*p* = 0.001, Fig 6F, seed-dressing plants excepted). The results for roots 0.2–1.0 mm in diameter were similar (*p* = 0.029 with *B*. *bassiana* and *p* = 0.006 with *M*. *brunneum*). No differences between inoculation methods and the control treatment were found in roots thicker than 1.0 mm (*p* = 0.154 with *B*. *bassiana* and *p* = 0.091 with *M*. *brunneum*; Fig 6E and 6F).

#### Nutrient contents of above-ground biomass

Table 2 and S3 Table show the dry weight and nutrient contents of the above-ground biomass from the sorghum plants at the end of the cropping period (93 DAS). Although the sorghum plants exhibited Fe deficiency symptoms (internerval yellowing throughout the trial) and purple leaf coloration due to P deficiency (in this case, only during the first two weeks), all the plants exhibited vigorous growth.

**Table 2:** Analysis of variance for above-ground plant dry weights and micronutrient contents in the biomass of the sorghum plants (mean ± standard error, *n* = 4) at the end of the experiment (93 DAS) with each fungus and inoculation method.

	Dry weight (g)	Fe (mg kg^-1^)	Mn (mg kg^-1^)	Zn (mg kg^-1^)	Cu (mg kg^-1^)
***B*. *bassiana***					
Seed dressing	1.59±0.12	31.3±2.5ab	10.2±0.2a	7.4±0.5	6.0±0.3a
Soil treatment	1.63±0.03	33.2±1.5a	9.3±0.6ab	8.4±0.3	6.2±0.1a
Leaf spraying	1.82±0.09	25.6±1.4b	8.2±0.3bc	8.7±1.2	5.4±0.1b
Control	1.62±0.19	18.2±1.9c	7.8±0.2c	9.7±0.5	4.9±0.2b
*p value*	0.531	<0.001	0.002	0.206	0.002
***M*. *brunneum***					
Seed dressing	1.60±0.12	29.8±1.3a	13.2±1.3a	8.4±0.4b	6.4±0.1a
Soil treatment	1.79±0.11	27.7±2.0a	12.2±0.7a	7.9±0.2b	5.6±0.1b
Leaf spraying	1.52±0.05	27.4±1.6a	11.0±0.7a	7.9±0.4b	5.4±0.5b
Control	1.62±0.19	18.2±1.9b	7.8±0.2b	9.7±0.5a	4.9±0.2b
*p value*	0.491	0.002	0.004	0.023	0.010

Different letters indicate significant differences between the levels of each factor according to the LSD *post hoc* test at *p* <0.05

There were no significant differences in dry weight, or in the K, P, Ca or Mg contents, of the above-ground plant biomass between the inoculation methods and the control treatment with either fungal strain (S3 Table). Overall, the nutrient contents fell within their normal ranges [61]. Inoculation with *B*. *bassiana* significantly altered the Fe, Mn and Cu contents (*p* < 0.001, *p* = 0.002 and *p* = 0.002, respectively) in relation to the control plants (Table 2). The mean Fe, Mn and Cu contents with soil treatment and seed dressing were higher than those for the leaf spraying and control groups. Inoculation with *M*. *brunneum* also altered the Fe, Mn, Cu and Zn contents to a significant extent (*p* = 0.002, *p* = 0.004, *p* = 0.023 and *p* = 0.010, respectively) (Table 2). Thus, the Fe and Mn contents of the inoculated plants were significantly higher than the contents of the control plants. However, the differences in Cu contents were only significant with seed dressing, which led to the highest levels relative to the control treatment. By contrast, the Zn content was slightly lower in all the inoculated plants than in the control plants.

Finally, the Fe_DTPA_ at the end of crop, which is a measure of Fe bioavailability, was not significantly affected by inoculation with either fungus. The mean Fe_DTPA_ contents (mg Fe kg^-1^) with *B*. *bassiana* (*p* = 0.056) were 1.16 ± 0.03 with soil treatment, 1.29 ± 0.03 with seed dressing and 1.28 ± 0.03 with leaf spraying, whereas those obtained with *M*. *brunneum* (*p* = 0.208) were 1.16 ± 0.02 with soil treatment, 1.20 ± 0.05 with seed dressing and 1.24 ± 0.03 with leaf spraying. The Fe_DTPA_ for the control group was 1.28 ± 0.04.

## Discussion

### *In vitro* experiment

The soil pH is one of the primary factors influencing the EPF growth and persistence in the soil [1]. The optimum pH for fungal growth is 5–10 for *B*. *bassiana* [62,63], approximately 7 for some *Metarhizium* species [64,65], and approximately 5.8 for some *Isaria* species [66]. These differences in pH values may explain why the three EPF strains performed differently depending on the particular combination of Fe sources and on the presence or absence of CaCO_3_. In fact, the pH of the culture medium ranged from 5.6 without CaCO_3_ to 8.4 with CaCO_3_ when using hematite or goethite as the Fe sources, and from 5.2 without CaCO_3_ to 6.8 with CaCO_3_ when ferrihydrite was the Fe source. In the last case, the amorphous nature of ferrihydrite caused it to release protons continuously while crystallizing [41]. This release might be the reason why ferrihydrite led to a pH of 6.8 and the other Fe sources led to a pH of 8 in the presence of CaCO_3_.

Fungi (EPF included) are known to alter their environment by releasing organic acids [67], and the concentrations of nutrients such as Fe, Cu, and Ag [68], Zn [69] or P [70] increase as a result. The released organic acids lower the pH of the medium and increase the solubility of Fe oxides. In our experiment, *B*. *bassiana* and *I*. *farinosa* basically raised the pH of the medium (Table 1) in relation to the control treatment without fungus in the absence of CaCO_3_. This increase in pH can be ascribed to the presence of Fe(NO_3_)_3_ and NaNO_3_ in the Czapek-Dox culture medium, providing NO_3_^–^ anions as a source of N and forcing the fungi to release OH^–^ ions, which had to be absorbed to ensure homeostatic regulation, thereby alkalizing the medium [71].

*Metarhizium brunneum* reduced the pH of the medium under calcareous conditions (i.e., in the presence of CaCO_3_). However, it is clear (Fig 2B) that this reduction did not occur in the absence of CaCO_3_, probably because the solubility of Fe was not reduced by the low prevailing pH, or with ferrihydrite as an Fe source in the presence of CaCO_3_, conditions under which Fe was more readily available, more reactive and soluble, because of the higher surface area of ferrihydrite [20,72]. In this situation, the fungi were required to produce smaller amounts of organic acids than under calcareous conditions or when the Fe source was a crystalline Fe oxide, and iron was thus less readily available.

In addition, fungi produce and release substances with low molecular weights called siderophores. These substances have a high affinity for Fe, and also for other nutrients such as Mn, Zn and Cu, which facilitates its chelation and makes it more readily available [27,28,73–75]. This Fe acquisition mechanism, which requires no direct alteration of the pH, may have compounded with the release of organic acids to increase the Fe availability in the culture medium.

As expected, the presence of CaCO_3_ reduced the Fe availability and raised the pH [76]. The three EPF strains increased the Fe availability in a different way; thus, *M*. *brunneum* probably released organic acids in more under calcareous conditions, and probably all three fungi produced siderophores. Based on the results for the three EPF strains and the wider pH range for the optimal growth of *B*. *bassiana*, we chose to use this species together with *M*. *brunneum* in the pot experiment under calcareous conditions.

### Pot experiment

Our calcareous artificial substrate mimicked the conditions of low Fe availability in calcareous soils and was useful for reducing the number of variables, potentially modulating the effect of EPF on the plant growth and Fe nutrition in these soils. Although the number of fungal propagules (CFU) of *B*. *bassiana* and *M*. *brunneum* decreased gradually throughout the pot experiment, it remained above the natural background level for the substrates in which the fungi were applied to soil at ≤ 10^4^ conidia g^-1^ [77]. The CFU in the substrate with soil treatment was higher for *M*. *brunneum* than it was for *B*. *bassiana*. This result is consistent with those of previous experiments [78,79] in which the increased size of the *M*. *brunneum* conidia resulted in easier retention in sandy substrates, whereas the smaller size of the *B*. *bassiana* conidia facilitated their vertical dragging by water through macropores in sand particles. Both *M*. *brunneum* and *B*. *bassiana* are rhizosphere-competent fungi [80] and can persist in the soil for a long time [81]. The CFU in the substrate with seed dressing may have resulted from fungal growth from the surface and inside of seeds and plants having been more marked with *M*. *brunneum* (CFU > 10^4^ conidia g^-1^ in the four samplings). Although the substrates were covered with aluminum foil to prevent contamination, leaf spraying led to CFU being detected in the substrate during the sampling following the application of the fungi. Nevertheless, the sterilization of the substrate implies that the microbial diversity in our sterile artificial substrate was not the same as it was in non-sterile substrates or under natural conditions, and this environment should have facilitated the growth of the EPF [82–85]. Consequently, the results obtained using a sterile substrate might not be comparable to those using the same substrate when it is non-sterile. The primary reason is the antagonistic effect of soil sterilization on substrate microbial diversity, which minimizes biological competence for EPF. However, similar trends in CFU in soil have been found in experiments that were developed by our research group using non-sterilized calcareous soils and *M*. *brunneum* applied to the surface [15].

Although *B*. *bassiana* is seemingly a more efficient endophyte than *M*. *brunneum* [86], our results do not support this assumption. In fact, *M*. *brunneum* was re-isolated to a greater extent from most plant tissues and in most samplings. The differences in persistence between *M*. *brunneum* and *B*. *bassiana* after inoculation may have resulted from the inoculation method, fungal strain, host plant genotype and sandy substrate in use. In fact, the re-isolation of *B*. *bassiana* from the sorghum plants inoculated by seed dressing, soil treatment and leaf spraying was previously found to be strongly influenced by the particular substrate (viz., sterile soil, non-sterile soil or vermiculite) [38]. Consequently, these factors influenced the success of the plant-endophyte associations (i.e., the re-isolation percentage) [87].

Our re-isolation results are consistent with those of previous studies in which *B*. *bassiana* and *M*. *brunneum* successfully colonized various plant tissues as endophytes, both in wheat inoculated with the two fungal strains [8] and in cassava roots inoculated with *B*. *bassiana* and *M*. *anisopliae* [88]. As suggested by Tefera et al. [38] for pot-grown sorghum, a successful colonization may have relied on the hyphal motion and growth in the internal structures of the plants. Additionally, as suggested by Quesada-Moraga et al. [45] for *Papaver somniferum*, and by Landa et al. [86], García et al. [89] and Resquín-Romero [90] for tomato, melon and alfalfa plants, the re-isolation of the fungus at a fairly long distance from the inoculation point indicates that the circulation of the endophyte in a plant is due to a passive movement within the xylem and to the development of hyphae in vascular elements.

The plant inoculation method was also critical in the pot experiment because it governed the colonization of the plant tissues during the cropping period and had a differential effect on the plant growth (plant height and root) and LCC. Consistent with previous results [38], the three inoculation methods (viz., seed dressing, soil treatment and leaf spraying) enabled the colonization of the leaves, stems and roots of the sorghum plants. The re-isolation results were similar to those obtained previously in similar assays [40], in which they found leaf spraying and soil treatment to lead to endophytic colonization by 80% in bean plants (*Phaseolus vulgaris* cv. Calima) that were inoculated with *B*. *bassiana*. However, the extent of colonization was dependent on the particular plant tissue. In previous studies, the leaves and stems responded better to leaf spraying treatments in which the fungi were applied to the above-ground plant biomass, whereas the roots were more responsive to soil treatments in which the fungi were applied to the soil [40].

The two fungal strains and three inoculation methods used here had no effect on the dry weight of the above-ground plant biomass or plant roots; in addition, as in other trials [91,92], the fungal application did not impair the plant growth. However, the effect on the plant height was dependent on the particular inoculation method and especially marked at the end of the experiment in the plants inoculated by seed dressing with *B*. *bassiana* and those inoculated by soil treatment or seed dressing with *M*. *brunneum*. By contrast, leaf spraying with *B*. *bassiana* decreased the final plant height while increasing the number of leaves compared to the other three treatments with *M*. *brunneum*. The increase in plant growth was not reflected in the above-ground plant biomass, probably because the pots were filled with only 250 g of substrate. Our results are consistent with those of [3], who found that some species in the *Metarhizium* and *Beauveria* genera commonly colonized the rhizosphere to promote plant growth; with those of [7], who reported an increase in leaf density in field corn inoculated with *M*. *anisopliae* via seed dressing; and with those of Sasan and Bidochka [4], who found *M*. *robertsii* to promote root growth in switch grass inoculated using the same method.

The significant positive effect of inoculation on LCC at 72 DAS (with both fungi and the three inoculation methods) and 79 DAS (soil treatment with both fungi, and soil treatment and leaf spraying with *M*. *brunneum*), that is, when the Fe chlorosis symptoms in the control plants peaked and the LCC was lowest, reflects a clear-cut beneficial effect. The interaction of the LCC × plant height (an indicator of plant health status with respect to the Fe chlorosis) behaved similarly and led to more marked differences between the inoculated plants and the control group. The positive effect on the sorghum plants was more persistent with *M*. *brunneum* (from 72 to 87 DAS but only 72 and 79 DAS with the three inoculation methods) than it was with *B*. *bassiana* (from 72 to 79 DAS but only 72 DAS with all three inoculation methods). The inoculation method was also the key variable in the persistence of the effect on the LCC; thus, soil treatment and leaf spraying had a similar effect at 72 and 79 DAS, whereas seed dressing with *B*. *bassiana* was significantly influential at only 72 DAS. With *M*. *brunneum*, the three inoculation methods had a similar positive effect at 72 and 79 DAS, but only the soil treatment extended the effect to 87 DAS. Therefore, inoculation with *B*. *bassiana* or *M*. *brunneum* increased the LCC in young leaves and increased the Fe content, irrespective of the inoculation method, in the above-ground biomass of sorghum plants grown under calcareous conditions at the end of the pot experiment. In summary, inoculation had a positive effect on the Fe nutrition. These results are consistent with those of the *in vitro* assay, in which the EPF increased the bioavailability of Fe from ferrihydrite in the calcareous medium. In addition, they are consistent with those of Sánchez-Rodríguez et al. [14], who found that applying *B*. *bassiana* to wheat and tomato seeds grown in an artificial calcareous substrate to increase the LCC to an extent that was dependent on the ferrihydrite content of the substrate; and with those of Sánchez-Rodríguez et al. [15], who found that applying *M*. *brunneum* to calcareous soils to alleviate the Fe chlorosis symptoms in sorghum and wheat plants.

Overall, the three inoculation methods were effective at promoting root growth in relation to the control group, with the soil treatment being the most effective method in terms of the SRL, SRA and fine roots with both fungi. The fine roots (less than 0.2 mm in diameter), SRA and total root length were related to the ability of the plants to explore the soil and obtain water and nutrients from it [93–96]. These findings are highly significant for the uptake of nutrients such as Fe, Mn and Cu by plants, especially when they are scant. Our results are consistent with those of Jaber and Erkerly [97], who found that applying *M*. *brunneum* and *B*. *bassiana* via seed dressing would systematically colonize different parts of bean plants (*Vicia faba*), and they increased the root lengths 7 days after inoculation, thereby resulting in improved plant growth (plant height, number of leaves, fresh root weight and shoot weight) at 14 and 28 days after inoculation.

Soil treatment was the inoculation method that produced the largest amounts of CFU with both fungi, but particularly *M*. *brunneum*, in our experiment. This result may have influenced the re-isolation of the fungi, which was similar to that observed with seed dressing and greater with *M*. *brunneum* than with *B*. *bassiana* in the roots. Liao et al. [12] used corn (*Zea mays*), and Parsa et al. [98] used common bean (*Phaseolus vulgaris*), and they both found a beneficial effect of *Metarhizium* spp. on the plant growth and root promotion to rely on the success of the fungus-plant association. Their results suggest that physical root colonization is a prerequisite for many of the beneficial effects of these fungi, which could partly explain our results. Additionally, Liao et al. [12] found that the *Metarhizium* strains used in their study could promote plant growth and auxin production. Some evidence also likely exists that may explain why soil treatment was the most efficient inoculation method. Thus, as reported by Mukherjee et al. [99] and Harman et al. [100] for the *Trichoderma* species, we believe that the mechanisms by which *Metarhizium* stimulates plant growth are probably multifactorial and include the promotion of plant growth through antibiotic, antiparasitic and host resistance-inducing effects. In any case, our insect-free pot experiment revealed that the EPFs studied here had direct positive effects on their plant hosts.

In general, the mineral element concentrations fell within the normal ranges [61]. An overall increase in the Fe, Mn and Cu mean contents of the above-ground plant biomass in the inoculated plants relative to the control plants was observed (especially with seed dressing and soil treatment). By contrast, inoculation with the fungi slightly decreased the Zn contents. This detrimental effect on Zn may have resulted from the potential adsorption of the metal onto Fe oxides, diminishing its uptake by plants [101]. We hypothesize that the alterations in root parameters (total root length and increased SRL, SRA and fine roots) in inoculated plants facilitated the exploration of the substrate, thereby resulting in increased contents of micronutrients (primarily Fe) and had positive effects on the LCC and plant growth (in the form of plant height) in our Fe-limiting calcareous substrate.

## Conclusions

The three EPF strains used in the *in vitro* assay increased the Fe availability under different conditions with respect to the Fe source (three Fe oxides of different crystallinities and solubilities) and growing substrate (calcareous or non-calcareous). *Metarhizium brunneum* was the most effective fungus, seemingly as a result of its increase of the Fe availability by releasing organic acids to lower the pH of the calcareous media. Both *B*. *bassiana* and *M*. *brunneum* alleviated the Fe chlorosis symptoms in inoculated sorghum plants grown on an artificial calcareous substrate. Although the three inoculation methods used in this experiment had positive effects on the plants, the soil treatment was the most effective method. Thus, it resulted in the increased persistence of the fungus in the substrate (CFU), re-isolation from different plant tissues to an extent comparable to that of seed dressing, a more persistent effect on the chlorophyll concentration of young leaves and plant growth relative to the other two inoculation methods, and a similar ability to increase the Fe uptake by sorghum plants. As revealed by the root parameter values, the soil treatment also resulted in a better exploration of the substrate. Therefore, the inoculation method is a key factor to consider in designing sustainable agricultural strategies based on EPF. The results of this work expand on the existing knowledge of plant-EPF-soil relations, and they underscore the importance of the inoculation method and the use of EPF to improve plant Fe nutrition in calcareous substrates beyond their role as biocontrol agents.

## Supporting information

S1 Table Mean FeDTPA and pH. Fe_DTPA_ and pH (mean ± standard error, *n* = 4) of the culture medium after 35 days of fungal growth in the presence of different sources of Fe and the presence or absence of calcium carbonate.
(DOCX)

S2 Table Chlorophyll total concentration (CTC). Chlorophyll total concentration (CTC) extracted from the two youngest leaves of each plant (mean ± standard error, *n* = 4) according to the combination of fungus and inoculation method at the end of the experiment (93 DAS).
(DOCX)

S3 Table Total macronutrient contents of plant biomass and root parameters. Analysis of variance of macronutrient contents in the above-ground biomass, root dry weight and root length of sorghum (mean ± standard error, *n* = 4) as a function of the fungus and inoculation method at the end of the experiment (93 DAS).
(DOCX)

S1 Fig Number of leaves. Number of plant leaves for each treatment (mean ± standard error, *n* = 4) applied to *B*. *bassiana* (A) and *M*. *brunneum* (B). Different letters indicate significant differences between different levels of each factor according to an LSD *post hoc* test at *p* <0.05.
(TIF)

S2 Fig Images of the roots of plants that were inoculated with the fungi by using different inoculation methods. (TIF)
