# Supplementary material for: Entomopathogenic fungi-based mechanisms for improved Fe nutrition in sorghum plants grown on calcareous substrates
Source: PLoS One. 2017 Oct 5;12(10):e0185903. doi: 10.1371/journal.pone.0185903 (PMC5628914; doi:10.1371/journal.pone.0185903)
Supplement: S1 Table — FeDTPA and pH (mean ± standard error, n = 4) of the culture medium after 35 days of fungal growth in the presence of different sources of Fe and the presence or absence of calcium carbonate. (DOCX) [file pone.0185903.s001.docx]

| **S1 Table.** Fe_DTPA_ and pH (mean ± standard error, *n* = 4) 0f the culture medium after 35 days of fungal growth in the presence of different sources of Fe (0 or 250 mg L^-1^) and the presence or absence of CaCO_3_ (0 or 300 mg L^-1^). | | | | | | | |
| --- | --- | --- | --- | --- | --- | --- | --- |
|  | **With CaCO_3_** | | |  | **Without CaCO_3_** | | |
|  | **Fe_DTPA_** |  | **pH_KCl_** |  | **Fe_DTPA_** |  | **pH_KCl_** |
|  | **(mg L^-1^)** |  |  |  | **(mg L^-1^)** |  |  |
| **No Fungus** |  |  |  |  |  |  |  |
| Control^(a)^ | 0.0 ± 0.0c |  | 8.4 ± 0.1a |  | 0.0 ± 0.0b |  | 5.2 ± 0.1b |
| Ferrihydrite | 2.0 ± 0.2a |  | 6.8 ± 0.0b |  | 5.1 ± 0.4a |  | 5.2 ± 0.0b |
| Hematite | 0.3 ± 0.0bc |  | 8.4 ± 0.0a |  | 0.4 ± 0.1b |  | 5.7 ± 0.1a |
| Goethite | 0.5 ± 0.1b |  | 8.4 ± 0.1a |  | 0.4 ± 0.0b |  | 5.4 ± 0.0ab |
| *p* value | <0.001 |  | <0.001 |  | <0.001 |  | 0.008 |
|  |  |  |  |  |  |  |  |
| ***B. bassiana*** |  |  |  |  |  |  |  |
| Control^(a)^ | 0.0 ± 0.0b |  | 7.9 ± 0.0c |  | 0.0 ± 0.0c |  | 7.5 ± 0.3b |
| Ferrihydrite | 20.2 ± 2.7a |  | 8.5 ± 0.0a |  | 32.3 ± 1.2a |  | 8.5 ± 0.0a |
| Hematite | 3.7 ± 0.1b |  | 8.3 ± 0.1b |  | 9.7 ± 1.0b |  | 7.9 ± 0.1b |
| Goethite | 3.7 ± 0.1b |  | 8.0 ± 0.1c |  | 7.4 ± 0.7b |  | 8.0 ± 0.2ab |
| *p* value | <0.001 |  | <0.001 |  | <0.001 |  | 0.014 |
|  |  |  |  |  |  |  |  |
| ***I. farinosa*** |  |  |  |  |  |  |  |
| Control^(a)^ | 0.0 ± 0.0c |  | 8.4 ± 0.1ab |  | 0.0 ± 0.0c |  | 7.3 ± 1.0 |
| Ferrihydrite | 24.2 ± 1.5a |  | 8.5 ± 0.1a |  | 29.7 ± 1.4a |  | 8.3 ± 0.1 |
| Hematite | 8.0 ± 0.6b |  | 8.3 ± 0.1bc |  | 14.5 ± 1.2b |  | 8.3 ± 0.0 |
| Goethite | 7.6 ± 0.4b |  | 8.3 ± 0.0c |  | 13.6 ± 0.7b |  | 8.0 ± 0.1 |
| *p* value | <0.001 |  | 0.023 |  | <0.001 |  | 0.491 |
|  |  |  |  |  |  |  |  |
| ***M. brunneum*** |  |  |  |  |  |  |  |
| Control^(a)^ | 0.0 ± 0.0c |  | 6.5 ± 0.4b |  | 0.0 ± 0.0c |  | 4.8 ± 0.1d |
| Ferrihydrite | 32.5 ± 3.3a |  | 7.3 ± 0.3a |  | 94.6 ± 17.5a |  | 6.8 ± 0.1a |
| Hematite | 14.6 ± 2.4b |  | 7.1 ± 0.1ab |  | 34.8 ± 1.6b |  | 6.4 ± 0.0b |
| Goethite | 11.6 ± 6.3bc |  | 6.8 ± 0.3ab |  | 28.7 ± 7.3bc |  | 6.0 ± 0.0c |
| *p* value | <0.001 |  | 0.203 |  | <0.001 |  | <0.001 |
| ^(a)^ Control (0 mgFe L^-1^) | | | | | | | |
