# Supplementary material for: Entomopathogenic fungi-based mechanisms for improved Fe nutrition in sorghum plants grown on calcareous substrates
Source: PLoS One. 2017 Oct 5;12(10):e0185903. doi: 10.1371/journal.pone.0185903 (PMC5628914; doi:10.1371/journal.pone.0185903)
Supplement: S2 Table — Chlorophyll total concentration (CTC) extracted from the two youngest leaves of each plant (mean ± standard error, n = 4) according to the combination of fungus and inoculation method at the end of the experiment (93 DAS). (DOCX) [file pone.0185903.s002.docx]

| **S2 Table.** Chlorophyll total concentration (CTC) extracted from the two youngest leaves of sorghum plants (mean ± standard error, *n* = 4) according to the combination of fungus and inoculation method at the end of the experiment (93 DAS). | | | |
| --- | --- | --- | --- |
|  | ***B. bassiana*** |  | ***M. brunneum*** |
|  | **CTC (µg cm^–2^)** |  | **CTC (µg cm^–2^)** |
| Seed dressing | 19.4±2.0ab |  | 16.6±1.1bc |
| Soil treatment | 21.8±2.5a |  | 19.0±1.5ab |
| Leaf spraying | 14.3±1.4b |  | 21.5±1.3a |
| Control | 14.6±0.9b |  | 14.6±0.9c |
| *p* value | 0.029 |  | 0.011 |
| Different letters indicate significant differences between the levels of each factor according to the LSD *post hoc* test at *p* <0.05 | | | |
