# Supplementary material for: Entomopathogenic fungi-based mechanisms for improved Fe nutrition in sorghum plants grown on calcareous substrates
Source: PLoS One. 2017 Oct 5;12(10):e0185903. doi: 10.1371/journal.pone.0185903 (PMC5628914; doi:10.1371/journal.pone.0185903)
Supplement: S3 Table — Analysis of variance of macronutrient contents in the above-ground biomass, root dry weight and root length of sorghum (mean ± standard error, n = 4) as a function of the fungus and inoculation method at the end of the experiment (93 DAS). (DOCX) [file pone.0185903.s003.docx]

| **S3 Table.** Analysis of variance for macronutrients contents in the above-ground plant biomass, root dry weight and root length of sorghum plants (mean ± standard error, *n* = 4) as a function of the fungus and inoculation method at the end of the experiment (93 DAS). | | | | | | | | | | | |
| --- | --- | --- | --- | --- | --- | --- | --- | --- | --- | --- | --- |
|  | **Above-ground plant biomass** | | | | | | | | **Root** | | |
| ***B. bassiana*** | **K** |  | **P** |  | **Ca** |  | **Mg** |  | **Root dry weight** |  | **Root length** |
|  | **(g kg^-1^)** |  | **(g kg^-1^)** |  | **(g kg^-1^)** |  | **(g kg^-1^)** |  | **(g)** |  | **(m)** |
| Seed dressing | 40.1±3.1 |  | 2.93±0.35 |  | 3.9±0.4 |  | 4.0±0.3 |  | 0.65±0.08 |  | 21.15±2.39a |
| Soil treatment | 36.5±2.5 |  | 2.92±0.42 |  | 4.1±0.0 |  | 4.1±0.2 |  | 0.82±0.11 |  | 21.59±1.37a |
| Leaf spraying | 32.2±3.2 |  | 2.10±0.05 |  | 4.3±0.3 |  | 3.2±0.1 |  | 1.00±0.09 |  | 22.53±2.75a |
| Control | 39.9±1.7 |  | 2.95±0.26 |  | 4.0±0.3 |  | 3.7±0.3 |  | 0.83±0.07 |  | 12.16±1.26b |
| *p* value | 0.188 |  | 0.187 |  | 0.775 |  | 0.137 |  | 0.106 |  | 0.031 |
| ***M. brunneum*** |  |  |  |  |  |  |  |  |  |  |  |
| Seed dressing | 37.5±2.1 |  | 2.69±0.29 |  | 4.0±0.3 |  | 3.5±0.3 |  | 0.90±0.08 |  | 18.21±2.67bc |
| Soil treatment | 36.0±1.7 |  | 2.33±0.22 |  | 3.6±0.3 |  | 3.0±0.0 |  | 0.80±0.11 |  | 26.54±0.81a |
| Leaf spraying | 34.7±2.0 |  | 2.48±0.12 |  | 3.8±0.2 |  | 3.1±0.1 |  | 0.92±0.06 |  | 21.34±2.27ab |
| Control | 39.9±1.7 |  | 2.95±0.26 |  | 4.0±0.3 |  | 3.7±0.3 |  | 0.83±0.07 |  | 12.16±1.26c |
| *p* value | 0.278 |  | 0.560 |  | 0.647 |  | 0.136 |  | 0.728 |  | 0.004 |
| Different letters indicate significant differences between the levels of each factor according to the LSD *post hoc* test at *p* <0.05 | | | | | | | | | | | |
