# Supplementary figures and images for: Entomopathogenic fungi-based mechanisms for improved Fe nutrition in sorghum plants grown on calcareous substrates
Source: PLoS One. 2017 Oct 5;12(10):e0185903. doi: 10.1371/journal.pone.0185903 (PMC5628914; doi:10.1371/journal.pone.0185903)

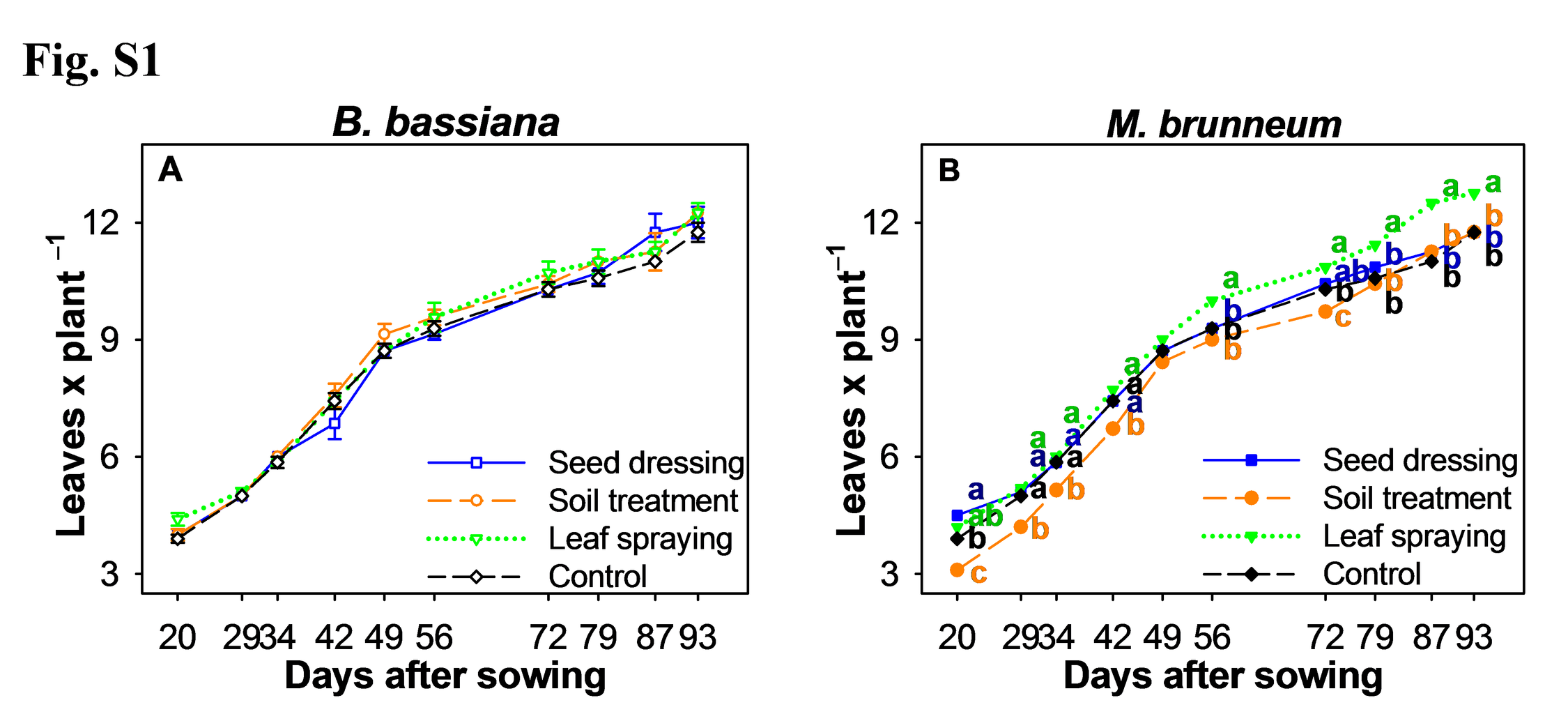

Supplement: S1 Fig — Number of plant leaves for each treatment (mean ± standard error, n = 4) applied to B. bassiana (A) and M. brunneum (B). Different letters indicate significant differences between different levels of each factor according to an LSD post hoc test at p <0.05. (TIF) [file pone.0185903.s004.tif]

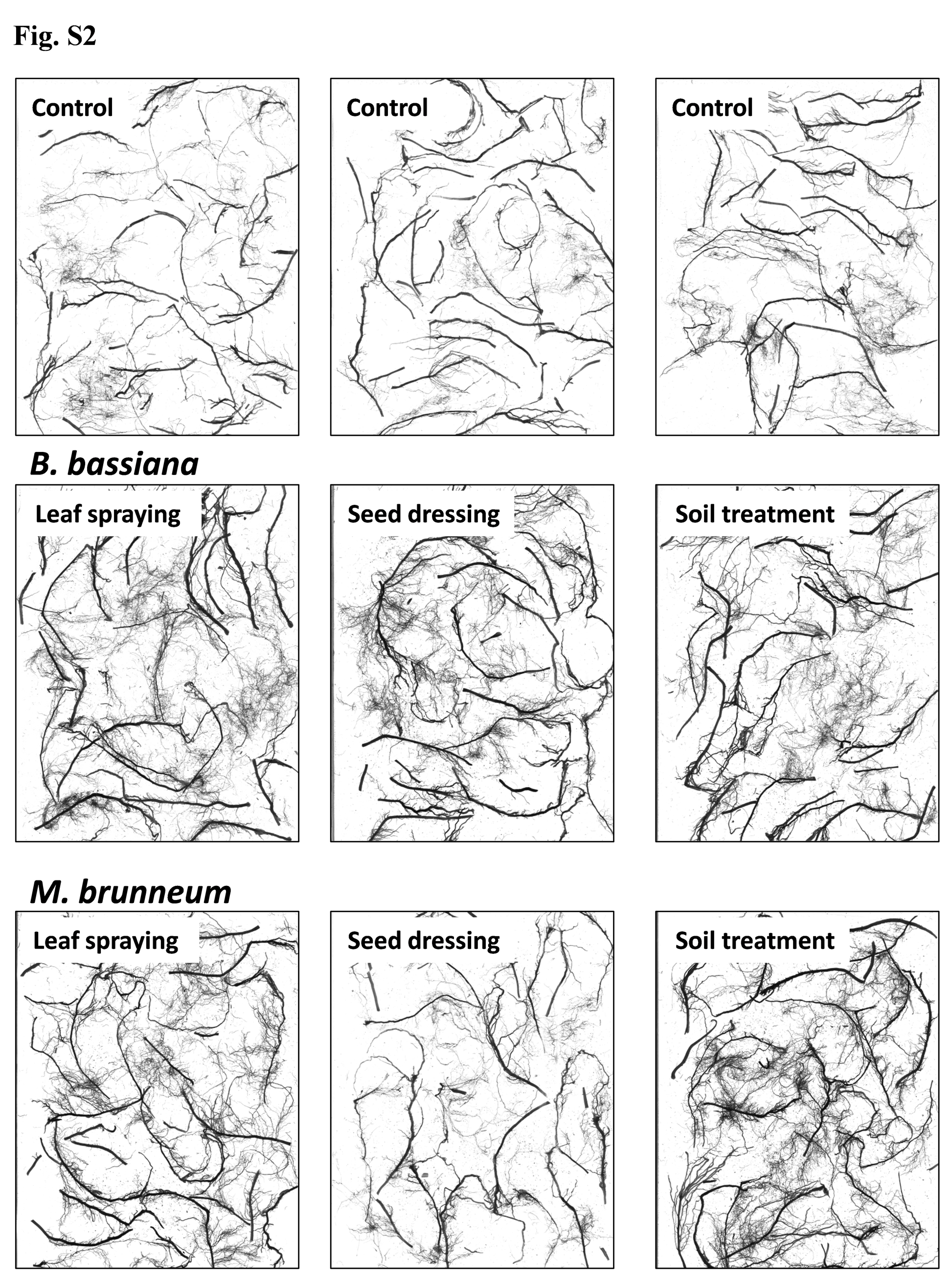

Supplement: S2 Fig — (TIF) [file pone.0185903.s005.tif]
